# Clinical utility of urine drug screening in children younger than 7 years in the pediatric emergency department: a multicenter retrospective study

**DOI:** 10.3389/fped.2026.1859634

**Published:** 2026-07-28

**Authors:** Renana Gelernter, Eran Kozer, Vered Hugi-Shitrit, Danna Krupik, Or Moskovich, Nitai Levy, Jordanna H. Koppel, Yael Lurie

**Affiliations:** 1 Department of Pediatric Emergency Medicine, Shamir Medical Center, Zrifin, Israel; 2 Gray Faculty of Medical and Health Sciences, Tel Aviv University, Tel Aviv, Israel; 3 Department of Pediatric Emergency Medicine, Ziv Medical Center, Safed, Israel; 4 Department of Pediatric Emergency Medicine, Rappaport Faculty of Medicine, Haifa, Israel; 5 Technion – Israel Institute of Technology, Haifa, Israel; 6 Department of Pediatric Emergency Medicine, The Edmond and Lily Safra Children’s Hospital, Chaim Sheba Medical Center, Ramat Gan, Israel; 7 Israel Poison Information Center, Clinical Pharmacology and Toxicology Section, Rambam Health Care Campus, Haifa, Israel

**Keywords:** cannabis, child protection, emergency medicine, pediatrics, urine drug screen (UDS)

## Abstract

**Background:**

Urine drug screening (UDS) is an immunoassay frequently used in emergency departments to detect drug exposure; however, its clinical utility remains debated. This study aimed to describe the clinical and epidemiological characteristics of young children who underwent UDS and to assess whether the test results influenced case management.

**Methods:**

We conducted a multicenter retrospective chart review across four hospitals, including children younger than 7 years who underwent UDS between 2010 and 2023.

**Results:**

Among the 693 patients (mean age, 30.7 ± 19.4 months; 59.1% male), 115 UDS tests were positive. In 36 patients (5.2%), the UDS contributed to establishing the diagnosis. Tetrahydrocannabinol (THC) was the most common substance identified in these cases. Hospitalization was more common in the UDS-established diagnosis group (33/36, 91.7% vs. 494/657, 75.2%; *P* = 0.026), and involvement in child protective services was also more common (26/36, 72.2% vs. 53/657, 8.1%; *P* < 0.001). The hospitalization duration was shorter in these patients (2.6 ± 2.1 vs. 4.2 ± 6.4 days; *P* = 0.002), and electroencephalography was performed less often (4/36, 11.1% vs. 229/655, 35.0%; *P* = 0.003).

**Conclusions:**

UDS influences the diagnosis and management of approximately 5% of young children, most commonly when THC is detected. Further studies are needed to refine the role of UDS in the pediatric emergency department.

## 1 Introduction

Urine drug screening (UDS) is an immunoassay frequently employed in the emergency department (ED) to detect common prescription drugs and drugs of abuse. Because of their limited sensitivity and specificity, positive and negative screening tests do not provide definitive confirmation or exclusion of poisoning. In adults, the available literature does not support the routine use of UDS for acute management in the ED (1–5). Similar findings have been reported in pediatric populations (6–8). However, those studies included a wide age range, including adolescents, and some used more comprehensive analytic tests.

The utility of UDS may be greater in young children (9–11). In this age group, a positive result may prompt the involvement of child protective services. This intervention is critical, as specialized therapeutic and educational programs for families with confirmed cases of child maltreatment have been shown to significantly lower maltreatment recidivism (12). UDS may also contribute to the diagnosis of apparent life-threatening events (13–16). Furthermore, positive toxicological findings can guide targeted management and prevent unnecessary or invasive diagnostic evaluations, such as lumbar punctures, computed tomography (CT) scans, and electroencephalograms (EEGs). However, data on the clinical utility of UDS in young children are limited. Prior pediatric research has largely been limited to narrow clinical scenarios (13, 14, 16) or specific substance classes (11, 15). Moreover, the few studies evaluating physician decision-making have either included older pediatric cohorts (9) or relied on comprehensive, laboratory-based analytical testing rather than rapid emergency department immunoassays (10). Objectively identifying these exposures has become increasingly important as the availability landscape shifts; for instance, while recreational cannabis remains illegal under Israeli law, highly regulated medical cannabis prescriptions have become more prevalent, elevating the risk of accidental household exposure in this vulnerable age group. The aim of this study was to investigate the clinical and epidemiological characteristics of young children for whom UDS was performed and to examine whether toxicology screening results influenced case management.

## 2 Methods

### 2.1 Study design and patients

We conducted a multicenter retrospective chart review in the pediatric emergency departments of four academic hospitals. Three hospitals were large tertiary centers, and one was a smaller hospital. The study was approved by the institutional review board at each participating site. Because this was a retrospective review with no identifiable information and no effect on patient care, the local Helsinki Committee of the principal investigator's hospital granted an exemption from informed consent. Approval was granted by the Ethich committee all participating medical centers (0147-21-ASF; 0067-21-ZIV; SMC-D-9579-22).

### 2.2 Data collection

We reviewed the medical records of all pediatric patients younger than 7 years who underwent UDS during ED evaluation or hospitalization as part of their workup between January 2010 and October 2023. For hospital D, data were available only from 2016 to 2020.

We recorded demographic characteristics, chief complaints, laboratory workup results, imaging findings, diagnostic procedures, medical interventions, and clinical outcomes via an Excel study datasheet. The immunoassays used at the participating hospitals during the study period are listed in Supplementary Table S1. All hospitals were screened for amphetamines, barbiturates, benzodiazepines, cannabinoids, cocaine, 3,4-methylenedioxymethamphetamine (ecstasy), methadone, morphine/opiates, and tricyclic antidepressants. Some immunoassays also screened for oxycodone, fentanyl, synthetic opioids, methamphetamine, and phencyclidine.

While this standard panel predominantly targets illicit substances and drugs of abuse, it also encompasses routinely prescribed therapeutic agents (e.g., benzodiazepines and tricyclic antidepressants) and drugs with high abuse potential (oxycodone, fentanyl).

### 2.3 Case definitions

The cases were classified as follows:

1. UDS for establishing a diagnosis:
  - In cases with no history of exposure, the test was ordered because of clinical suspicion and yielded a positive result consistent with the clinical presentation.
  - In cases with reported exposure, the substance history was unclear, and the test identified a substance consistent with the patient's clinical symptoms.
2. The UDS does not establish a diagnosis:
  - Patients with a clear history of exposure to a specific substance when the diagnosis was established independently of the test result.
  - Patients with no history of exposure in which the test was ordered because of clinical suspicion and was negative.
  - Patients with positive UDS after prehospital or emergency department treatment, such as positive benzodiazepine results after diazepam administration for seizure control.

To ensure consistency across sites and minimize classification bias, initial case definitions and data extraction were performed by a senior pediatric emergency physician at each participating center, following detailed, structured instructions provided by the principal investigator (PI) (RG). The PI then conducted a centralized review of the baseline clinical data for all cases. Cases demonstrating inconsistencies or borderline classifications were resolved via interactive discussion and consensus-based adjudication between the site investigator and the PI, ensuring a uniform application of the study definitions across the cohort.

To evaluate the impact on clinical management, specific resource utilization endpoints were defined a priori: hospitalization, length of stay, involvement of child protective services, and the performance of diagnostic investigations (lumbar puncture, brain imaging, electroencephalography, and abdominal ultrasound). The frequencies of these *a priori* outcomes were compared directly between the UDS-established diagnosis and non-established diagnosis cohorts.

### 2.4 Statistical analysis

Descriptive statistics were used to summarize the study population. Associations between categorical variables were assessed via the chi-square test or Fisher's exact test, as appropriate. Continuous variables were compared via t tests or Mann–Whitney *U* tests, as appropriate. Missing data were not imputed. Analyses were performed on available data, and denominators reflect the number of patients with non-missing values for each variable. Statistical significance was set at *P* < 0.05. Analyses were performed via SPSS version 25 (IBM Corp., Armonk, NY).

## 3 Results

**Figure 1:**
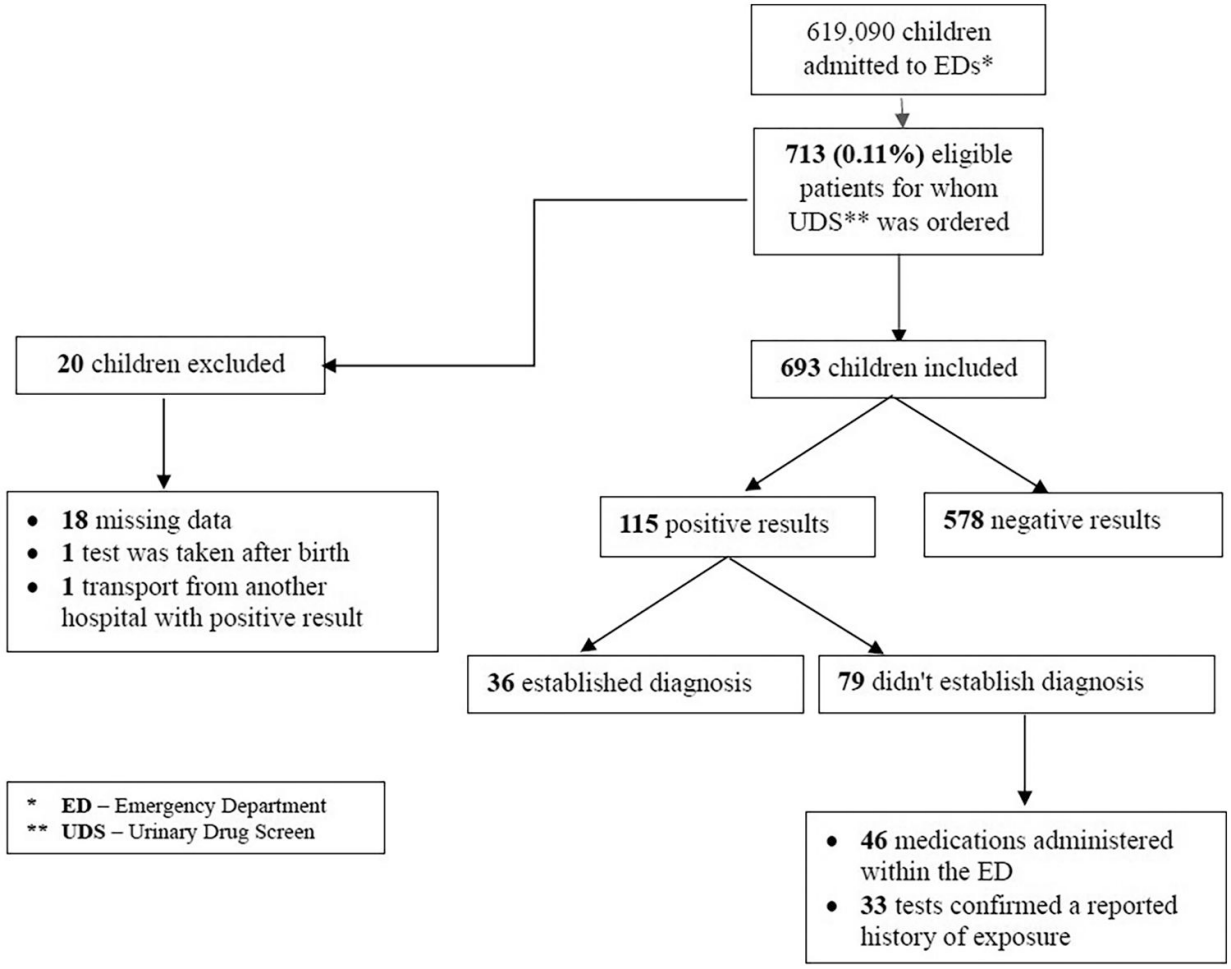
Study flow diagram.

During the study period, 619,090 children younger than 7 years presented to the emergency departments of the participating hospitals. UDS was ordered for 713 patients (0.11%). Twenty cases were excluded: 18 because of missing data, one because the UDS was performed immediately after birth, and one because the UDS result was already known on arrival. The final analysis included 693 cases (Figure 1). Patient characteristics are presented in Table 1.Owing to incomplete documentation in the medical records, data were missing for some variables. The number of patients with available data is indicated for each variable and in the relevant tables. No imputation was performed; all analyses used available (complete-case) data.

**Table 1:** Characteristics of pediatric patients who underwent urine drug screening (*n* = 693).

Variable	Category	Value
Hospital	A	244/209,221 (0.12%)
	B	123/162,165 (0.07%)
	C	303/213,143 (0.14%)
	D	23/34,561 (0.07%)
Age, months	Mean ± SD	30.7 ± 19.4
Male sex	*n* (%)	407 (59.1%)
Ethnicity	Jewish	484 (69.8%)
	Arab	166 (24%)
	Other	43 (6.2%)
Indication for UDS	Neurological symptoms	575 (83.1%)
	History of exposure	86 (12.4%)
	Unusual parent behavior	4 (0.6%)
	Other	27 (3.9%)
Hospitalization	*n* (%)	527 (76.5%)
Length of hospitalization	Days, mean ± SD	4.1 ± 6.2

Among the 693 UDS tests, 115 (16.6%) yielded positive results. In one test, two drugs were detected, so there were 116 positive identifications in total. The UDS results are presented in Table 2.

**Table 2:** Urine drug screening results (*n* = 693).

Outcome	No. of cases (%)
Any positive result	115 (16.6%)
Drug identified (*n* = 116)	
Benzodiazepines	56 (48.2%)
Tetrahydrocannabinol (THC)	29 (25.0%)
Barbiturates	13 (11.2%)
Amphetamine	4 (3.4%)
Methamphetamine	2 (1.7%)
Cocaine	3 (2.6%)
Tricyclic antidepressants	3 (2.6%)
3,4-Methylenedioxymethamphetamine (MDMA)	2 (1.7%)
Morphine/opiates	1 (0.8%)
Oxycodone	1 (0.8%)
Methadone	1 (0.8%)
Phencyclidines	1 (0.8%)

Among the 115 positive results, 46 (40.0%) were attributed to medications administered within the ED, with benzodiazepines being the most common (33/46, 71.7%). These tests were therefore classified as not establishing the diagnosis.

In 36 patients (5.2%), UDS was considered to have established the diagnosis. THC was the most common substance contributing to the diagnosis (20/36, 55.0%). The remaining 16 cases comprised benzodiazepines (*n* = 6), cocaine (*n* = 3), amphetamines (*n* = 3), 3,4-methylenedioxymethamphetamine (*n* = 2), methamphetamine (*n* = 1), and opioids (*n* = 1). Among the 86 patients with a clear history of exposure, 54 had negative results: in 21 patients, the suspected substance was not included in the UDS panel; in 7 patients, the substance was included but the result was negative; and in 26 patients, the exposure involved unknown substances.

The remaining 69 positive tests comprised 36 cases where the UDS uniquely established the diagnosis, and 33 cases where the positive test merely confirmed a known history of exposure (accounting for the 32 single-exposure and 1 dual-exposure positive cases within the 86 patients presenting with a reported history).

Among the demographic characteristics examined, Arab ethnicity was the only variable significantly associated with a UDS-established diagnosis (Table 3).

**Table 3:** Variables associated with the UDS establishing a diagnosis.

Variable	UDS established diagnosis (*n* = 36)	UDS did not establish diagnosis (*n* = 657)	*P*-value
Ethnicity, Arab	20 (55.5%)	146 (22.2%)	<0.001
Ethnicity, Jewish	14 (38.9%)	470 (71.5%)	
Ethnicity, Other	2 (5.5%)	41 (6%)	
Age, months	25.4 ± 17.7	31.0 ± 19.4	0.09
Sex, male	23 (63.9%)	384 (58.8%)	0.60
Sex, female	13 (36.1%)	269 (41.2%)	
Indication			0.36
Neurological symptoms	29 (80.5%)	546 (83.2%)	
History of exposure	7 (19.4%)	79 (12.0%)	
Unusual parent behavior	0 (0%)	4 (0.6%)	
Other	0 (0%)	27 (4.1%)	

When evaluating clinical management, patients whose diagnosis was established by the UDS had higher rates of hospitalization, although their length of stay was shorter, and EEG was performed less frequently than those in the non-established diagnosis group. No significant differences were observed regarding the performance of lumbar puncture, head imaging, or abdominal ultrasound (Table 4).

**Table 4:** Management decisions associated with UDS establishing diagnosis.

Variable	UDS established diagnosis (*n* = 36)	UDS did not establish diagnosis (*n* = 657)	*P*-value
Lumbar puncture	11 (30.5%)	191 (29.1%)	0.85
Head CT or MRI	13 (36.1%)	239 (36.4%)	1.00
Abdominal ultrasound	11 (30.5%)	154 (23.4%)	0.32
Electroencephalogram	4 (11.1%)	229 (34.9%)	0.003
Hospitalization	33 (91.7%)	494 (75.2%)	0.026
Child protective services	26 (72.2%)	53 (8.1%)	<0.001
Length of hospitalization	2.6 ± 2.1	4.2 ± 6.4	0.002

## 4 Discussion

In this study, urine drug screening (UDS) established a diagnosis in 5.2% of the children (36 out of 693). In these cases, the results significantly influenced clinical decision-making, as evidenced by higher hospitalization rates and increased involvement of child protective services. Interestingly, the duration of hospitalization was shorter for these children. This corresponds to the expected pharmacological clearance window of most detected substances, which typically resolve within 24 hours, and matches data from a systematic review reporting a mean hospital stay of 27 hours following accidental pediatric cannabis ingestion (17).

With respect to diagnostic investigations, children with a UDS-confirmed diagnosis underwent fewer EEG procedures, although no significant differences were found in the utilization of brain imaging, abdominal ultrasound, laboratory testing, or lumbar punctures. This likely reflects the urgent nature of pediatric emergency medicine; critical investigations such as lumbar punctures and neuroimaging are often initiated immediately upon presentation, before toxicology results are available. In contrast, an EEG is generally considered a nonemergent test and is thus less likely to be performed once a toxicological cause is identified in the acute phase.

The higher rate of UDS-established diagnoses observed among Arab children is notable, though its underlying causes are likely complex and multifactorial. This disparity could potentially be influenced by communication barriers during initial clinical triage, cultural factors, or differing patterns of substance availability and exposure risks. Because this retrospective study was not designed to evaluate these sociocultural or linguistic variables, these findings should be interpreted with caution and warrant dedicated prospective evaluation.

While a 5.2% diagnostic yield may seem modest, it represents a clinically meaningful rate that justifies maintaining a low threshold for ordering UDS in young children presenting with unexplained neurological symptoms, altered mental status, or atypical clinical presentations. Rather than universal screening, our findings support a targeted but aggressive screening strategy in the ED, ensuring that toxic exposures are not missed in non-verbal cohorts where history is withheld or unobtainable.

The substantial increase in Child Protective Services (CPS) involvement within the UDS-established diagnosis group (72.2% vs. 8.1%, *P* < 0.001) highlights how heavily toxicological screening drives social welfare referrals. While unconfirmed immunoassays carry a risk of false positives due to cross-reactivity, maintaining a low threshold for protective intervention is vital in young children. Specialized parenting and social support programs have been shown to significantly reduce child maltreatment recidivism (12), making the ED referral a critical step for long-term safety.

A notable finding in our cohort was the high rate of negative UDS results among children with a known history of exposure (54 out of 86 patients). As detailed in our results, nearly 40% of these negative tests occurred because the suspected substance was simply not included in the standard institutional immunoassay panel. This discrepancy offers vital practical advice for emergency clinicians: a negative screening test cannot rule out ingestion when clinical suspicion is high. This finding underscores the clinical need for hospitals to regularly update their routine UDS panels to encompass prevalent regional prescription medications and emerging synthetic drugs, or to utilize more comprehensive analytical testing when acute poisoning is strongly suspected.

Our findings align with those of previous smaller cohorts, indicating that UDS impacts pediatric management. Prior research has shown unexpected urine findings influencing management in 3% (8) and 1.8% (10) of cases, often involving young children and resulting in social welfare or law enforcement interventions. Similar patterns were observed in studies of pediatric out-of-hospital cardiac arrest, where unexpected substances were detected in 5.3% of cases, primarily infants (15). Furthermore, in children under 12 years of age presenting with altered mental status and no known history of ingestion, urine drug screening (UDS) provides a 4.8% diagnostic yield (9).

In our cohort, THC was the most prevalent substance (55% of positive cases). During the entire study framework and to date, recreational cannabis possession and commercial sale remain prohibited under Israeli law. However medical cannabis is prescribed under strict restrictions. The household presence of these prescribed medical products may represent another source of accidental exposure among young children. As cannabis legalization has expanded globally (18–21), the frequency of pediatric THC exposure is expected to rise, further increasing the clinical relevance of UDS in emergency settings.

## 5 Limitations

This study is subject to several limitations. As a retrospective chart review, data documentation may be incomplete. The lack of standardized testing kits may have resulted in the omission of certain substances, such as methamphetamines, potentially underestimating the true incidence of poisoning. Furthermore, positive results were not confirmed via gold-standard analytical methods (e.g., GC‒MS). However, in this young pediatric population, the risk of false positives due to cross-reactivity with medications is expected to be minimal. Although the 13-year study period introduces potential variations in clinical practice over time, no major regulatory or legal changes regarding substance policies occurred during this framework to significantly impact our findings. For hospital D, data were available only for a subset of the study period (2016–2020). However, this restricted timeframe is unlikely to have significantly influenced our findings, given that no major regulatory or legal changes regarding substance policies occurred during the study framework, and patients from this center comprised only a small fraction (3.3%) of the overall study cohort. The involvement of different site investigators for case classification introduces a possibility of classification bias and a formal inter-rater agreement analysis was not performed. However, this risk was minimized by providing structured guidelines to all investigators and implementing a secondary data validation review by the PI. Finally, the retrospective determination of whether a UDS has “established” a diagnosis may not fully capture the real-time nuances of clinical decision-making. Finally, because this study was conducted entirely within the Israeli healthcare and legal systems, the observed rates of child welfare involvement and substance profiles may not be fully generalizable to countries with different legal frameworks regarding cannabis availability or alternative child protection protocols.

## 6 Conclusions

The UDS influenced the diagnosis and clinical management of approximately 5% of young children in this study, with THC identified as the most common diagnostic substance. Given its documented impact on hospital disposition and social welfare intervention, physicians should consider utilizing the UDS in the evaluation of young children with unexplained emergency presentations, provided they exercise careful clinical judgment when interpreting the results.

## Data Availability

The original contributions presented in the study are included in the article/supplementary material, further inquiries can be directed to the corresponding author.
