# Supplementary material for: Clinical utility of urine drug screening in children younger than 7 years in the pediatric emergency department: a multicenter retrospective study
Source: Front Pediatr. 2026 Jul 28;14:1859634. doi: 10.3389/fped.2026.1859634 (PMC13457201; doi:10.3389/fped.2026.1859634)
Supplement: Supplementary file 1 [file Table1.docx]

**Supplementary Table S1**: Urine drug screen immunoassay details

| **Hospital** | **Year** | **Immunoassay** | **Medication and drug of abuse included in the assay** |
| --- | --- | --- | --- |
| 1 | 2010-2011 | Acro biotech, inc. | Methadone, Barbiturates, Benzodiazepines, Cocaine, Tricyclic antidepressants, Opiates, MDMA (Ectasy), Cannabis (THC), Amphetamine, Phencyclidine |
|  | 2012 | Abon, Inc. | Methadone, Barbiturates, Benzodiazepines, Cocaine, Tricyclic antidepressants, Opiates, MDMA (Ectasy), Cannabis (THC), Amphetamine, Metamphetamine, Phencyclidine |
|  | 2013-7/2016 | Innovacon, Inc. | Methadone, Barbiturates, Benzodiazepines, Cocaine, Tricyclic antidepressants, Opiates, MDMA (Ectasy), Cannabis (THC), Amphetamine, Metamphetamine |
|  | 8/2016-2023 | Acro biotech, inc. | Methadone, Barbiturates, Benzodiazepines, Cocaine, Tricyclic antidepressants. Opiates, MDMA (Ectasy), Cannabis (THC), Amphetamine, Phencyclidine |
| 2 | 2010-2023 | Thermo Scientific™ CEDIA® / DRI™ Drugs of Abuse Immunoassays  Thermo Fisher Scientific | Methadone, Barbiturates, Benzodiazepines, Cocaine, Tricyclic antidepressants, Opiates, MDMA (Ectasy), Cannabis (THC), Amphetamine, EDDP (methadone metabolite, Oxycodone, Buprenorphine,  Propoxyphene |
| 3 | 2010-2013 | BIO-RAD TOX (Bio-Rad Laboratories Israel) | Methadone, Barbiturates, Benzodiazepines, Cocaine, Tricyclic antidepressants, Opiates, MDMA (Ectasy), Cannabis (THC), Amphetamine, Metamphetamine |
|  | 2014-2020 | BIO-RAD TOX (Bio-Rad Laboratories Israel) | Methadone, Barbiturates, Benzodiazepines, Oxycodone, Cocaine,Tricyclic antidepressants |
|  | 2014-2020 | Thermo Fisher, Architect ci4100 (Abbott) | Opiates, MDMA (Ectasy), Cannabis (THC), Amphetamine/Metamphetamine |
|  | 2021-2023 | Thermo Fisher, Architect ci4100 (Abbott) | Methadone, Barbiturates, Benzodiazepines, Oxycodone, Cocaine, Tricyclic antidepressants,Opiates , MDMA (Ectasy), Cannabis (THC), Amphetamine/Metamphetamine, Fentanyl |
| 4 | 2016-2020 | BIO-RAD TOX (Bio-Rad Laboratories Israel) | Methadone, Barbiturates, Benzodiazepines, Oxycodone Cocaine, Tricyclic antidepressants. Opiates, MDMA (Ectasy), Cannabis (THC), Amphetamine, Metamphetamine, Phencyclidine, |
